# MoS_2_ with Stable Photoluminescence Enhancement under Stretching via Plasmonic Surface Lattice Resonance

**DOI:** 10.3390/nano11071698

**Published:** 2021-06-28

**Authors:** Yen-Ju Chiang, Tsan-Wen Lu, Pin-Ruei Huang, Shih-Yen Lin, Po-Tsung Lee

**Affiliations:** 1 Department of Photonics, College of Electrical and Computer Engineering, National Yang Ming Chiao Tung University, Rm. 401 CPT Building, 1001 Ta-Hsueh Road, Hsinchu 300093, Taiwan; steves090003@gmail.com (Y.-J.C.); ping621.di98g@g2.nctu.edu.tw (P.-R.H.); 2 Department of Photonics, College of Electrical and Computer Engineering, National Chiao Tung University, Rm. 401 CPT Building, 1001 Ta-Hsueh Road, Hsinchu 300093, Taiwan; 3 Research Center for Applied Sciences, Academia Sinica, No. 128, Sec. 2, Academia Rd, Taipei 11529, Taiwan; shihyen@gate.sinica.edu.tw

**Keywords:** transition metal dichalcogenides, surface plasmon resonance, PL enhancement

## Abstract

In this study, by combining a large-area MoS_2_ monolayer with silver plasmonic nanostructures in a deformable polydimethylsiloxane substrate, we theoretically and experimentally studied the photoluminescence (PL) enhancement of MoS_2_ by surface lattice resonance (SLR) modes of different silver plasmonic nanostructures. We also observed the stable PL enhancement of MoS_2_ by silver nanodisc arrays under differently applied stretching strains, caused by the mechanical holding effect of the MoS_2_ monolayer. We believe the results presented herein can guarantee the possibility of stably enhancing the light emission of transition metal dichalcogenides using SLR modes in a deformable platform.

## 1. Introduction

Widely studied layered materials usually consist of lamellas weakly attached by van der Waals force, while the atoms in each lamella are strongly bonded in each two-dimensional (2D) plane. A famous example of this type of layered material in nature is graphite [1,2]. Over the past decade, by developing different artificial extractions or growth strategies, researchers have been able to obtain single-layered graphene, as well as to fully investigate its strong mechanical strength [3], ultrahigh electron mobility [4], and high thermal conductivity [5] at room temperature. As the semiconductor analogy of graphene, transition metal dichalcogenides (TMDs) [6,7] have received extensive attention in recent years. The most significant feature of TMDs compared with graphene is the direct bandgaps, which are beneficial as the channels of electrical transistors [8,9] or optical gain media for efficient light emission [10,11,12]. TMDs composed of different elements have different bandgaps which lead to different visible light emissions. Because TMDs are extremely thin (<1 nm), they do not disturb the optical properties of the dielectric or metallic photonic structures integrated with them. TMDs without dangling bonds also prevent unnecessary interactions with integrated structures. The above features are crucial for an optical gain medium in an efficient light source. However, researchers are still concerned that the thinness of TMDs can limit their optical emissions. Therefore, recently, efficiently enhancing the light emissions of TMDs has become one of the important issues in the field.

To meet the above-mentioned requirement for TMDs, researchers can increase the carrier recombination rate of TMDs materially or increase their spontaneous emission rate and quantum efficiency by optical techniques. In the first approach, growing different TMDs to form heterojunctions [13] and superposing their optical emissions is feasible. Alternatively, one can create oxygen-bonding-based defects or cracks in chemically doped TMDs [14] and significantly enhance the optical emission from these sites. In the second approach, researchers usually apply different nanophotonic structures [15,16,17,18,19,20,21,22,23,24,25,26] to TMDs to dramatically increase the light–matter interactions of TMDs. For example, the use of different photonic crystal (PhC) structures [15,16,17] with photonic bands and bandgaps for guiding and locally confining optical waves can efficiently accumulate photons in spatial and temporal domains. By utilizing a PhC cavity [18,19,20] with a sufficiently high-quality factor, such enhancement can even realize lasers.

Additionally, using metallic structures with surface plasmonic resonance (SPR) [27] is also a suitable choice. SPR is the collective electron oscillation at the metal–dielectric interface, which typically presents an extremely strong electric field concentration. Such a local field concentration can effectively overlap with a sheet gain medium in spatial terms and produce a large Purcell factor for enhancing light–matter interactions. By encircling this interface to be a nanoparticle, the SPR turns into localized SPR (LSPR) [28] with stronger fields and Purcell factor. Furthermore, if we arrange these nanoparticles into lattices, the Fano resonance effect due to lattice diffraction turns the LSPR in each nanoparticle into surface lattice resonance (SLR) [29]. Generally, SLR has additionally enhanced field intensity and narrower spectral linewidth, while it can significantly tune the wavelength by changing the lattice parameters. In recent years, by combining different TMDs with various PhC and plasmonic nanostructures [30], researchers have successfully enhanced their light emissions of TMDs by more than one order [21,22,23,24,25,26]. However, most demonstrations have still been on hard substrates, while only a few reports [30] have studied the emission enhancement in a deformable platform. A further investigation on such emission enhancement under deformation is still missing. Therefore, in this study, by integrating different plasmonic nanostructures sustaining SLR modes with a MoS_2_ monolayer in a deformable carrier, we theoretically and experimentally investigate the light emission enhancements of MoS_2_ and their stabilities under different stretching strains.

## 2. Design and Simulation

**Figure 1:**
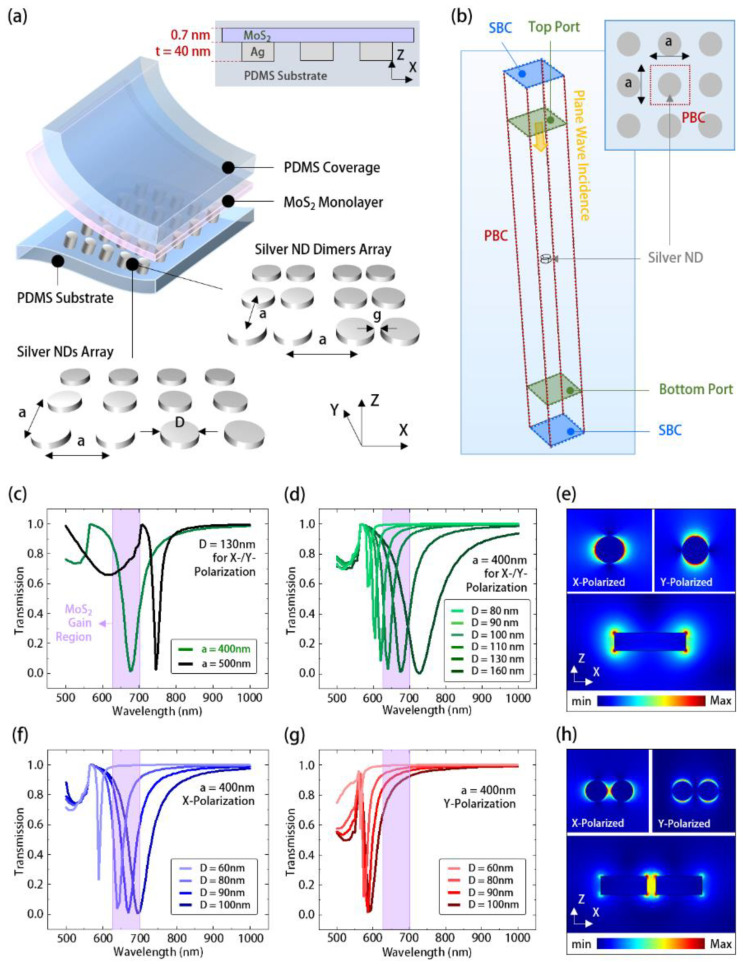
(**a**) Schematic and parameter definitions of silver ND and ND-dimer arrays covered with MoS_2_ monolayer in PDMS; (**b**) the lattice unit cell in the 3D FEM setup for simulating transmission spectra of silver ND arrays in PDMS; theoretical transmission spectra of silver ND arrays with different (**c**) *a* and (**d**) *D*, where the purple shadows represent the gain region of MoS_2_; (**e**) the electrical field distributions of the SLR mode along *XY* and *XZ* planes in silver ND array with *D* = 130 nm and *a* = 400 nm; theoretical transmission spectra of (**f**) *X*- and (**h**) *Y*-polarized SLR modes of silver ND-dimer array with different *D* values; (**g**) their electrical field distributions along *XY* and *XZ* planes under *D* = 100 nm, *g* = 20 nm, and *a* = 400 nm.

In this study, our proposed structure consists of silver nanostructures covered by a large-area MoS_2_ monolayer (with a thickness of 0.7 nm) in deformable polydimethylsiloxane (PDMS) substrate, as shown in Figure 1a. Herein, we use silver because of its lower optical absorption loss than gold and the ease of designing parameters of SLR for aligning with the gain spectrum of MoS_2_ (wavelength peak near 650 nm). We designed two different silver nanostructure topologies, including periodically arranged nanodiscs (NDs) and their dimers with a gap (*g*) along the *X* direction, as shown in the inset of Figure 1a. The insets also show their parameter definitions, including lattice constants (*a*) in the *X* and *Y* directions, ND diameter (*D*), and ND thickness (*t*).

To understand the optical properties of the above structure without MoS_2_, we utilized the three-dimensional (3D) finite element method (FEM, COMSOL, Burlington, MA, USA, Multiphysics software package) to characterize the theoretical transmission spectrum. Figure 1b shows the simulation lattice unit cell in the 3D FEM. In this setup, the silver ND unit cell embedded in PDMS had a cell size of *a* × *a* and was enclosed by top, bottom, and four side planes. The refractive index of PDMS is 1.405, and the dielectric function of silver is described by the Lorentz–Drude model [31,32]. The four side planes were set as the periodic boundary condition (PBC) to mimic the periodic ND array, and the top and bottom planes were set as a scattering boundary condition (SBC). We further inserted a top port above the ND to launch a broadband plane wave (essential for exciting the SLR mode) with different polarizations to the ND; a bottom port was set below the ND to receive and integrate the transmitted electric field for calculating the transmission spectra.

Figure 1c shows the theoretical transmission spectra of the silver ND arrays with lattice constants, *a,* from 400 to 500 nm and a fixed disc diameter, *D*, of 130 nm in PDMS. When the lattice constant decreased, the spectral valleys representing the SLR mode of the silver ND array showed a significant wavelength blue shift. This sensitivity to the lattice parameter is typical in SLR mode, in contrast to LSPR mode. Thus, aligning the SLR mode with the gain region of the MoS_2_ is feasible by tuning the lattice constant. In Figure 1e, the theoretical electric fields of *X*- and *Y*-polarized SLR modes along the *XY* and *XZ* planes concentrate along the top and bottom edges of the silver ND. In addition, because the ND is rotationally symmetric in topology, the *X*- and *Y*-polarized SLR modes showed the same transmission spectra and field distributions in Figure 1c,e, respectively. Furthermore, Figure 1d shows the theoretical transmission spectra of the silver ND arrays with different *D* values and fixed *a* of 400 nm. When the value of *D* increased from 80 to 160 nm, the SLR mode showed a wavelength redshift because of the elongated resonance path of LSPR in each ND. This wavelength dependence on *D* provides the other way to align the SLR mode with the MoS_2_ gain region.

In contrast, for the design of the silver ND-dimer array, owing to its different symmetry along the *X*- and *Y*-axis, the *X*- and *Y*-polarized SLR modes inside showed different spectral alignments with the MoS_2_ gain region, as shown in Figure 1f,g. This asymmetry led to significantly different field distributions of the *X*- and *Y*-polarized SLR modes, as shown in Figure 1h. However, owing to the dimer coupling effect, the *X*-polarized SLR mode profile, as shown in Figure 1h, still showed a much stronger field concentration within the gap than that of SLR in the silver ND array. Although the *Y*-polarized SLR mode did not effectively align in the spectrum with the MoS_2_ gain region, the strong field of the *X*-polarized SLR mode would still be beneficial for light emission enhancement. Moreover, to improve the above misalignment issue, without significantly changing the *X*-polarized SLR mode, one could further design elliptical NDs [33] to pull the *Y*-polarized SLR mode into the gain region.

## 3. Manufacturing Process

**Figure 2:**
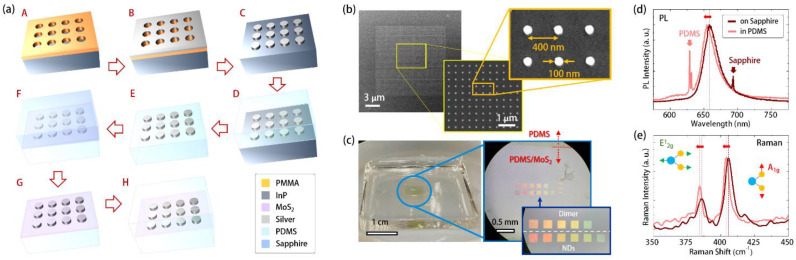
(**a**) Fabrication flowchart of the silver ND array covered by MoS_2_ monolayer in PDMS; (**b**) The top-view SEM images in different magnifications of silver ND array on InP substrate in Status C in (**a**); (**c**) picture and OM images of silver ND and ND-dimer arrays with covered MoS_2_ monolayer in a PDMS substrate; the measured (**d**) PL and (**e**) Raman spectra of MoS_2_ monolayer grown on sapphire and embedded in PDMS. The peaks on both sides of the MoS_2_ PL signal in (**d**) correspond to the emissions from PDMS and sapphire substrates.

Figure 2a shows the flowchart for manufacturing the above design. The process started by defining the plasmonic nanostructure patterns on the coated electron beam (*e*-beam) resist (polymethylmethacrylate, PMMA) on an InP substrate, using *e*-beam lithography (Status A). After evaporating the silver (Status B) and lifting off the patterned PMMA (Status C), we obtained the silver ND arrays on the InP substrate. Figure 2b shows the scanning electron microscope (SEM) images in different magnifications of a silver ND array on an InP substrate. Afterward, we spin coated PDMS (Sylgard184 of Dow Corning, a mixture of SylgardA and SylgardB with a volume ratio of 10:1) on the silver ND array and baked it at 60 °C for 12 h (Status D), and then removed the InP substrate using an HCl wet etching process at room temperature (Status E). Next, we prepared a wafer-scale MoS_2_ monolayer grown on a sapphire substrate by radio frequency sputtering [34] (see Supplementary Note S1 and Figure S1). After dicing, we covered a piece of MoS_2_ grown on sapphire (0.5 × 0.5 cm) on the exposed silver NDs (Status F). Owing to the viscosity of PDMS and the difference in hydrophilicity between MoS_2_ and sapphire, we could remove the sapphire substrate by the liquid wedging method [35] and uniformly attached the above diced MoS_2_ monolayer on the silver ND array (Status G). Eventually, we realized our proposed structure by sealing the MoS_2_ and silver ND array using PDMS. The above sealing step results in a symmetric structure of the MoS_2_/ND array combination and prevents the silver from oxidation by air.

Figure 2c shows the picture and optical microscope (OM) images of the silver ND and ND-dimer arrays covered with MoS_2_ in PDMS and manufactured by the above process. The picture clearly shows the uniformly attached MoS_2_ monolayer in the center of the PDMS substrate. The different disc diameters in each silver ND and ND-dimer arrays resulted in wavelength shifts of SLR modes, which reflected in different colors of each array in Figure 2c. To ensure the quality of MoS_2_ after the above manufacturing process, using a 532 nm laser excitation, we obtained the photoluminescence (PL) and Raman scattering spectra of MoS_2_ before and after embedding in the PDMS. In Figure 2d, the PL emissions from the MoS_2_ showed slight blue shifts (~4 nm) in wavelength, while the Raman peaks in Figure 2e corresponded to A^1^_g_ and E^1^_2g_ vibration mode shifts from 386 cm^−1^ and 406 cm^−1^ to 384 cm^−1^ and 404 cm^−1^, respectively. These slight peak shifts in PL and Raman spectra come from the strain relaxation [36] of MoS_2_ after transfer. Nevertheless, the invariant Raman peak difference (~20 cm^−1^) between A^1^_g_ and E^1^_2g_ vibration modes still guaranteed the monolayer feature of MoS_2_ after transfer.

## 4. Results and Discussions

### 4.1. PL Enhancement of MoS_2_ via Silver ND and ND-Dimer Arrays in PDMS

**Figure 3:**
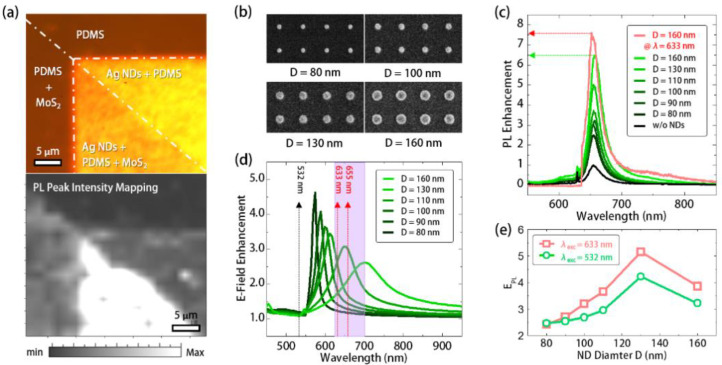
(**a**) (top) The OM image and (bottom) PL peak intensity mapping (*λ_em_* = 655 nm) of sample areas with different material combinations; (**b**) the SEM images of silver ND arrays with *D* from 80 to 160 nm; (**c**) the measured PL spectra of MoS_2_ covering these silver ND arrays in PDMS. The PL spectrum of MoS_2_ with silver ND array with *D* = 160 nm under a 633 nm laser excitation is also in the same plot; (**d**) theoretical enhancement of electrical field of SLR modes within MoS_2_ in silver ND arrays under different *D* values and wavelengths; (**e**) the calculated *E_PL_* contributed by SLR modes in silver ND arrays with different *D* values under *λ_exc_* of 532 and 633 nm.

In measurement, we use a 532 nm continuous wave laser to excite the manufactured structures at room temperature. First, Figure 3a shows the OM image of the silver ND array partially covered with a fractured MoS_2_ monolayer. It includes areas with different material combinations by silver ND array, MoS_2_ monolayer, and PDMS. The measured PL intensity spatial mapping in Figure 3a clearly shows the enhanced PL emission of MoS_2_ by the silver ND array. By this initial confirmation, we further characterized the MoS_2_ with silver ND arrays with *D* values from 80 to 160 nm, whose OM and top-view SEM images are shown in Figure 2c and Figure 3b, respectively. In Figure 3c, the PL enhancement of MoS_2_ increased with *D* and reached an enhancement value of 6.5 when *D* was 160 nm. The PL enhancement increased with increases in *D*, which was mainly attributed to the spectral alignment between the SLR mode and MoS_2_ gain region, as the prediction in Figure 1b. More specifically, the PL enhancement *E_PL_* is related to the excitation laser absorption and Purcell factor of the utilized SLR mode [22]. We can express the *E_PL_* as the summation of electrical field enhancements *E’*(*λ_exc_*)/*E*(*λ_exc_*) and *E’*(*λ_em_*)/*E*(*λ_em_*) of SLR mode. *E’*(*λ_exc_*)/*E*(*λ_exc_*) is the ratio of the electric field within the MoS_2_ layer with and without silver ND array at the excitation wavelength *λ_exc_* and *E’*(*λ_em_*)/*E*(*λ_em_*) is with the same definition at the emission wavelength *λ_em_*. *E’*(*λ_exc_*)/*E*(*λ_exc_*) represents the enhancement in laser absorption by the SLR mode at *λ_exc_*. *E’*(*λ_em_*)/*E*(*λ_em_*) approximates the Purcell factor [37] of the SLR mode, which expresses the enhancement of radiative spontaneous emission rate by the SLR mode at *λ_em_*.

In our FEM simulation, by integrating the electric field of SLR mode within the MoS_2_ layer, we calculated the electric field enhancements (compared with the electric field in the MoS_2_ layer without silver ND array) provided by silver ND arrays with different D values at different wavelengths, as shown in Figure 3d. With *λ_exc_* and *λ_em_* of 532 and 655 nm, respectively, the E_PL_ estimated from Figure 3d and recorded in Figure 3e increased with D, which approximately agreed with the measured PL enhancement. It should be noted that there is a mismatch between the *D* for maximum enhancement occurrence in the experiment and simulation. It has been reported that this mismatch is the result of approximating the Purcell factor by the electric field of SLR mode (i.e., *E’*(*λ_em_*)/*E*(*λ_em_*) we used above) in evaluating the PL enhancement [37]. In addition, according to the definition of *E_PL_*, we can further enhance the PL emission by enlarging the term *E*’(*λ_exc_*)/*E*(*λ_exc_*), which represents the excitation absorption. In Figure 3c, by using a 633 nm continuous wave laser excitation, we can obtain a higher PL enhancement value of 7.6 under *D* = 160 nm. The other cases with different *D* values all show stronger PL emissions under 633 nm laser excitation than those under *λ_exc_* of 532 nm (see Supplementary Note S2 and Figure S2).

**Figure 4:**
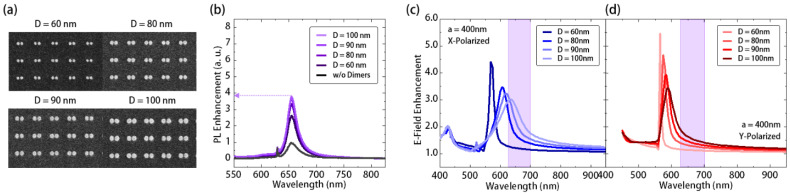
(**a**) The SEM images of silver ND-dimer arrays with *D* values from 60 to 100 nm; (**b**) the measured PL spectra of MoS_2_ covering these silver ND arrays after embedding in PDMS; theoretical electrical field enhancement of (**c**) *X*- and (**d**) *Y*-polarized SLR modes under different wavelengths in silver ND-dimer arrays with different *D*.

For comparison, we further characterized the silver ND-dimer array under the same excitation condition (*λ_exc_* = 532 nm). The OM and top-view SEM images are shown in Figure 2c and Figure 4a, respectively. Figure 4b shows that, in the experiments, we observed the same tendency towards an enhancement of PL from the array of ND dimers as from the ND array. As we mentioned before, according to the definition of *E_PL_*, the total electric field coupled in the MoS_2_ layer dominates the PL enhancement instead of the local maximum electric field. Therefore, even though the ND dimer showed an SLR local electric field over two times stronger than in a single ND, their similar total electric fields still led to similar PL enhancements. As we discussed, related to Figure 1c, the low total electric field in the ND dimer came from different symmetries of *X*- and *Y*-polarized SLR modes. The calculated *E_PL_*, shown in Figure 4c, agreed with the above prediction. Specifically, in Figure 4c, the *E_PL_* provided by *X*-polarized SLR mode at *D* = 100 nm matched well with the gain region of MoS_2_. In contrast, the *Y*-polarized SLR mode showed misalignment with *λ_em_* and contributed less *E_PL_* than the *X*-polarized SLR mode. If one would like to improve the PL enhancement of the silver ND-dimer array, as we discussed before, shaping the discs to be elliptical along the Y-axis could shift the Y-polarized SLR mode to a longer wavelength for aligning with the MoS_2_ gain region. However, in providing similar PL enhancements, the ND with a simpler topology and larger manufacturing tolerance than the present ND dimer would be a better choice.

### 4.2. PL Enhancement of MoS_2_ with ND and ND-Dimer Arrays under Stretching

**Figure 5:**
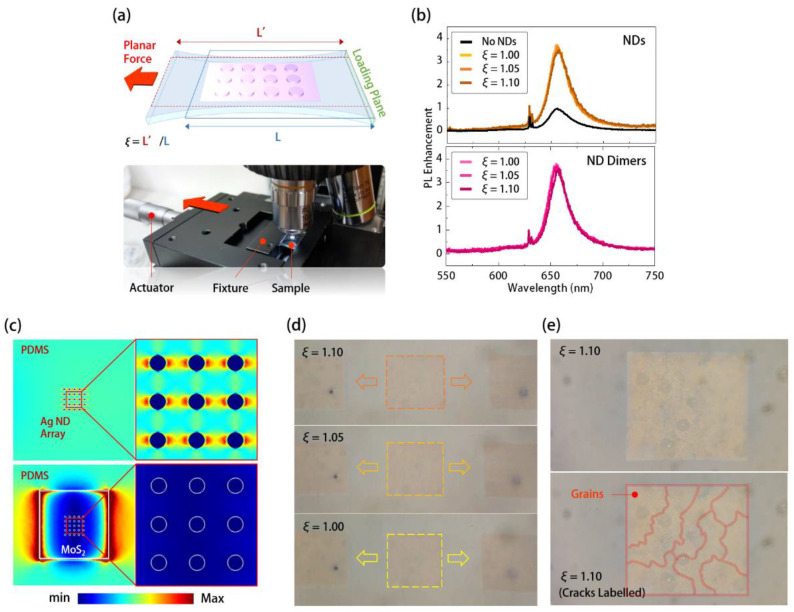
(**a**) (top) Schematics of stretching sample in measurement and simulation, definition of stretching strain *ξ*, and (bottom) picture of the stretching stage in PL and Raman spectra measurement system; (**b**) the measured PL spectra of the MoS_2_ covered with silver ND (top, *D* = 110 nm) and ND-dimer (bottom, *D* = 100 nm) arrays in PDMS under different stretching strains *ξ* from 1.0 to 1.1; (**c**) theoretical strain distributions along the planes of the silver ND arrays with (bottom) and without (top) MoS_2_ coverage in PDMS under an applied *ξ* of 1.1 along the *X*-axis; (**d**) the OM images of silver ND array covered with MoS_2_ under the applied *ξ* from 1.0 to 1.1; (**e**) high magnification OM images of MoS_2_ (with and without crack labels) with silver ND array under an applied *ξ* of 1.1 along the *X*-axis.

To further study the PL enhancements of MoS_2_ by silver ND and ND-dimer arrays under deformation in experiments, we fixed the sample on a custom-designed stretching stage in Figure 5a and stretched it along a specific direction. The stretching stage consists of fixtures and a linear actuator, and its applied strain *ξ* definition is the length ratio of the sample after (*L’*) and before (*L*) stretching, as illustrated in Figure 5a. Using this setup, we applied stretching strain along the *X* direction to the MoS_2_ in PDMS with silver ND and ND-dimer arrays. Figure 5b shows their measured PL spectra under stretching *ξ* from 1.0 to 1.1. Their peak wavelengths were almost invariant, while the PL intensities only showed less than 7% degradation under *ξ* of 1.1. However, the PL enhancement was very sensitive to the lattice expansion of the silver ND array (see Supplementary Figure S3). A reasonable explanation for the above slight PL variation could be the low strain transfer of MoS_2_ in PDMS under stretching, due to the huge stiffness difference between MoS_2_ and PDMS. In this case, the silver ND and ND-dimer arrays beneath are “held” by the MoS_2_ and become immobilized under stretching. Therefore, the SLR electric field enhancements in the silver ND and ND-dimer arrays are also stable under stretching, which produce the almost invariant PL enhancements shown in Figure 5b. In addition, it is also foreseeable that we could observe a similarly stable PL enhancement of MoS_2_ under stretching when replacing the present SLR plasmonic structure with the LSPR plasmonic structure that is not sensitive to lattice changes.

To verify the above prediction, we performed a mechanical simulation using 3D FEM. For the simulation, we set a loading plane in the simulation domain (PDMS substrate) and applied a planar force on the opposite plane to the MoS_2_ and silver ND array embedded inside, as illustrated in Figure 5a. We set the other four planes as loading free in all directions. The top of Figure 5c shows the theoretical strain distribution of the silver ND array in PDMS without MoS_2_ coverage under applied stretching stress along the *X*-axis. In this case, the strain nonuniformly concentrated in the PDMS regions between each silver ND. Such a nonuniform strain distribution is known as the film-edge induced strain [38] and comes from the stiffness difference between two neighboring materials. This strain distribution results in the lattice expansion of the ND array along the *X*-axis. Meanwhile, the almost zero strain distribution inside the silver NDs also means that there is no deformation of NDs under the applied stress. In contrast, in the case with MoS_2_ coverage, the bottom figure of Figure 5c shows that most strain distributed in the PDMS near the edge of MoS_2_, while there was almost no strain distribution between the silver NDs. This means the silver ND array beneath the MoS_2_ immobilized as predicted above, which led to an invariant SLR mode in stably enhancing the PL emission of MoS_2_ under stretching.

However, from the OM images in Figure 5d, we still observed that the ND arrays obviously elongated with stretching instead of being immobilized as we predicted above. This elongation is reversible when the applied stretching stress is released. To understand the contradiction between this phenomenon and our prediction above, we further examined the magnified OM image of ND arrays under *ξ* of 1.1, as shown in Figure 5e. In Figure 5e, the observed cracks along the *Y*-axis on the silver ND array could be responsible for the contradiction. In our study, because the area of MoS_2_ covering the silver ND array was quite large (100 × 100 μm^2^), the produced film-edge-induced strain near the MoS_2_ would be huge, even under low applied stress. Therefore, if certain structural asymmetries exist in the integration of silver ND array and MoS_2_, the induced strain concentrates near the asymmetry, which breaks the MoS_2_ into plural grain regions accompanied by the cracks labeled in Figure 5e. However, in this case, the induced strain was distributed along the cracks. Therefore, the silver ND array beneath each MoS_2_ grain still immobilized under stretching, as shown in Figure 5c, which still guaranteed the stable PL enhancements shown in Figure 5b.

## 5. Conclusions

In this study, based on silver ND and ND-dimer arrays covered by a MoS_2_ monolayer in a deformable PDMS substrate, we theoretically studied the PL enhancement of MoS_2_ by SLR modes within as well as the corresponding demonstration in experiments. When we further applied different stretching to the MoS_2_ with silver ND and ND-dimer arrays, the PL enhancements and the peak wavelengths of MoS_2_ still remained stable. In this case, because of the stiffness difference between MoS_2_ and PDMS, the MoS_2_ mechanically held the ND and ND-dimer arrays beneath the MoS_2_. This resulted in immobilized ND and ND-dimer arrays and SLR modes within and thus provided stable PL enhancement of MoS_2_ under stretching, even for the real case of large-area MoS_2_ cracks into plural grains. We believe the results presented herein provide an important reference for stably enhancing TMD light emission using SLR modes in a deforming platform.

## Supplementary Materials

The following are available online at https://www.mdpi.com/article/10.3390/nano11071698/s1, Figure S1: Preparation of large-area MoS_2_ monolayer on a sapphire substrate, Figure S2: PL enhancements of MoS_2_ with silver ND arrays under different excitations, Figure S3: Measured PL spectra from MoS_2_ covering with silver ND arrays with different D and lattice constants.
