# Supplementary material for: MoS2 with Stable Photoluminescence Enhancement under Stretching via Plasmonic Surface Lattice Resonance
Source: Nanomaterials (Basel). 2021 Jun 28;11(7):1698. doi: 10.3390/nano11071698 (PMC8307818; doi:10.3390/nano11071698)
Supplement: Supplementary file 1 [file nanomaterials-11-01698-s001.zip › nanomaterials-1248651-supplementary.pdf]

nanomaterials

Supplementary Materials

MoS_2 with Stable Photoluminescence Enhancement under Stretching via Plasmonic Surface Lattice Resonance

Yen-Ju Chiang [1,2], Tsan-Wen Lu [1,2,*], Pin-Ruei Huang [1,2], Shih-Yen Lin [3] and Po-Tsung Lee [1,2,*]

1 Department of Photonics, College of Electrical and Computer Engineering, National Yang Ming Chiao Tung University, Rm. 401 CPT Building, 1001 Ta-Hsueh Road, Hsinchu 300093, Taiwan; steves090003@gmail.com (Y.-J.C.); ping621.di98g@g2.nctu.edu.tw (P.-R.H.)
2 Department of Photonics, College of Electrical and Computer Engineering, National Chiao Tung University, Rm. 401 CPT Building, 1001 Ta-Hsueh Road, Hsinchu 300093, Taiwan
3 Research Center for Applied Sciences, Academia Sinica, No. 128, Sec. 2, Academia Rd, Taipei 11529, Taiwan; shihyen@gate.sinica.edu.tw
* Correspondence: tsanwenlu@gmail.com (T.-W.L.); potsung@mail.nctu.edu.tw (P.-T.L.)

Supplementary Note 1: Preparation of Large-Area Mos_2 Monolayer on a Sapphire Substrate

To prepare the MoS_2 film, at first, we deposit the molybdenum (Mo) metal on a sapphire substrate using a radio-frequency (RF) sputtering system. The metal deposition continues for 17 s under a 30 sccm argon (Ar) gas flow, while the RF power and background pressure are at 30 W and 5×10^{-3} Torr. Afterward, we place the sample with deposited Mo in a high-temperature furnace tube for further sulfurization. During the sulfurization procedure, we use Ar as the carrier gas with a flow rate of 200 sccm and place 0.25 g of sulfur powder upstream of the gas flow. The evaporation temperature for the sulfur powder is 160 °C, while the furnace pressure and growth temperature keep at 50 Torr and 850 °C, respectively. Figure S1 shows the picture and cross-sectional high-resolution transmission electron microscope (HRTEM) image of the MoS_2 film grown on a sapphire substrate via the above process. In Fig. S1, we can clearly observe its monolayer feature.

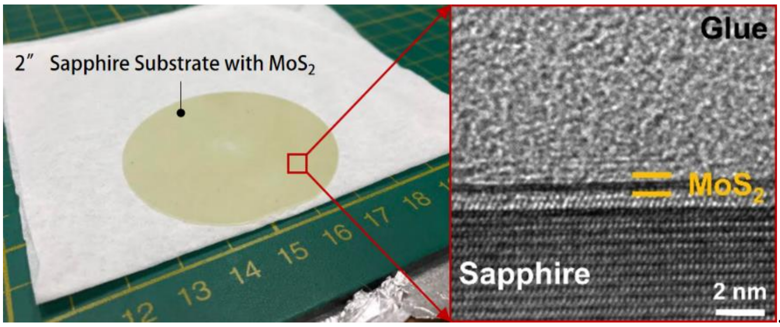

Figure S1. (left) Picture and (right) cross-sectional HRTEM image of the MoS_2 film grown on a sapphire substrate via the sputtering process.

Supplementary Note 2: PL Enhancements of MoS_2 with Silver ND Arrays under Different Excitations

Figures S2(a) and (b) show the measured PL enhancement and corresponding enhanced PL spectra from MoS_2 covering with silver ND arrays with different *D* from 80 to 160 nm under λ_{exc} = 488, 532, and 633 nm laser excitations. For the ND array with *D* = 90 nm, the SLR mode inside obviously misalign with the gain spectrum, as in the theoretical electrical field enhancement in Fig. S2(c). Besides, the SLR mode also misaligns with all the used λ_{exc}, which leads to very few $E'(\lambda_{exc})$ components and only about three times PL enhancement for all the excitation conditions. However, for the case with D of 110 nm, the PL enhancement under λ_{exc} of 633 nm is significantly stronger than those under λ_{exc} = 488 and 532 nm because the SLR mode tends to align with λ_{exc} = 633 nm and contributes more $E'(\lambda_{exc})$ components for PL enhancement. The same trend still exists in the case with *D* = 160 nm based on the same reason.

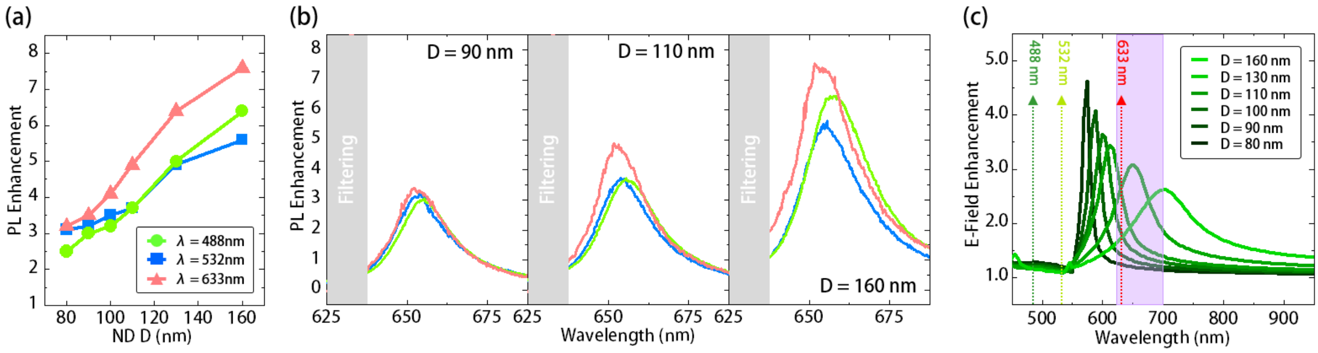

Figure S2. (**a**) The measured PL enhancement from MoS_2 covering with silver ND arrays with different *D* under different laser excitations. (**b**) The measured PL spectra from MoS_2 covering with silver ND arrays with *D* of 90, 110, and 160 nm under different laser excitations. (**c**) Theoretical electrical field enhancement by SLR modes in silver ND arrays with different *D*, as a function of wavelength.

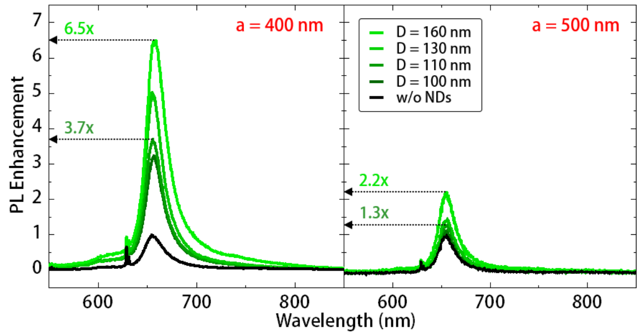

Figure S3. The measured PL spectra from MoS_2 covering with silver ND arrays with different *D* from 100 to 160 nm and lattice constants of (left) 400 and (right) 500 nm under a 532 nm laser excitation. For the silver ND arrays with *D* = 160 and 110 nm, when their lattice constants increase from 400 to 500 nm, the PL enhancements degrade from 6.5 and 3.7 to 2.2 and 1.3, respectively.
